# Comparison of Airborne SARS‐CoV‐2 Omicron and Pre‐Delta Variants Around Infected Patients

**DOI:** 10.1002/jmv.70258

**Published:** 2025-02-20

**Authors:** Carl‐Johan Fraenkel, Sara Thuresson, Patrik Medstrand, Malin Alsved, Jakob Löndahl

**Affiliations:** ^1^ Division of Infection Medicine, Department of Clinical Sciences Lund University Lund Sweden; ^2^ Department of Clinical Microbiology and Infection Prevention and Control Skåne University Hospital Lund Sweden; ^3^ Division of Ergonomics and Aerosol Technology, Department of Design Sciences Lund University Lund Sweden; ^4^ Department of Translational Medicine, Clinical Virology Lund University Lund Sweden

**Keywords:** aerosol, aerosol generating procedures, air, Omicron, SARS‐CoV‐2, transmission

## Abstract

Transmissibility has increased during the evolution of SARS‐CoV‐2, possibly by improved airborne transmission. An increased transmission was noted also in many hospitals. We analyzed SARS‐CoV‐2 in room air of hospitalized Omicron infected patients and compared results with previous findings with pre‐Delta variants to study if SARS‐CoV‐2 was more prevalent in patient rooms after the introduction of Omicron. Only 4 of 75 (5%) air samples, from 3 of 43 included patients, were positive during the early Omicron wave, compared to 14/120 (12%), from 10 of 60 included patients during the initial wave. No certain statistical difference between virus variants could be established, but the tendency was a lower occurrence at Omicron infected patients, also when adjusting for relevant confounders. These finding do not support the initial hypothesis that increased SARS‐CoV‐2 aerosol emission from diagnosed patients with Omicron could explain any increased risk of hospital transmission.

## 1 Background

The evolution of new variants of SARS‐CoV‐2 has been associated with ever increasing transmissibility, co‐evolving with antigenic drift to achieve immune evasion [1]. Increased transmissibility might be caused by improved viral shedding in aerosol particles, leading to an enhanced ability to spread through inhalation [2]. Studies have reported higher virus concentrations in exhaled breath in patients infected by different variants of concern compared to ancestral virus‐types [3, 4]. With the introduction of Omicron, a number of outbreaks were reported in which airborne transmission was considered the most likely mode of transmission and a significant further improvement in airborne transmission ability was suspected [5]. Further studies on exhaled air have reported a high shedding of Omicron variants in aerosols, but similar to Alpha and Delta variants [3, 4, 6, 7, 8]. A higher replication competence in the human bronchi and nasal tissues was proposed as a possible mechanism for increased virus‐containing aerosol formation [9]. The Omicron variant surge led to recommendations to further strengthen airborne precautions in hospitals [10]. Whether the proposed higher shedding of Omicron variants to aerosols also result in more frequent findings of SARS‐CoV‐2 in hospital air compared to the initial strains has, to the best of our knowledge, not been investigated.

We studied SARS‐CoV‐2 RNA in hospital air during the primary wave and at the beginning of the Alpha variant emergence and examined associations to factors possibly involved in production of virus‐containing aerosols in a previous study [11]. The aim of the present study was to analyze if SARS‐CoV‐2 RNA was more common in patient room air during the Omicron wave compared to the beginning of the COVID‐19 pandemic.

## 2 Materials and Methods

### 2.1 Design

An observational study of SARS‐CoV‐2 RNA presence in patient room air was performed at wards treating patients with COVID‐19 in one hospital in Skåne, southern Sweden, from January 2022 to May 2022. During the study period, SARS‐CoV‐2 Omicron BA.2 subvariant was dominant. Collected data included patient characteristics, such as the cycle threshold (Ct) value of the most recent nasopharyngeal swab containing SARS‐CoV‐2 and virus subvariant, as well as distance from patient to air sampler, room ventilation and ongoing potential aerosol generating procedures (AGP). Results were compared with air samples collected during the initial wave (March 20, 2020 to April 9, 2021), presented in a previous study [11]. To make the cohorts more comparable, we excluded patients treated at the Intensive care units (ICU) during the first wave since no included patient was treated at the ICU during the Omicron period. During the initial period no regular virus typing was performed, but 20 of a total 120 samples were collected during February to April 2021 when the Alpha variant was swiftly introduced. As the Delta variant emerged July 2021, samples collected during the initial period are defined as pre‐Delta.

### 2.2 Setting and Patients

Air samples were collected at five wards involved in COVID‐19 care. Standard ventilation was 3–4 air changes per hour (ACH), but some rooms had enhanced ventilation either by preinstalled 8 ACH mechanical ventilation or by mobile High‐Efficiency Particulate Air (HEPA)–filtration units, delivering filtered air at an approximate airflow rate of 200 L/s. All patients were laboratory confirmed with COVID‐19. Patients with recent SARS‐CoV‐2 PCR test were preferred. Data on patient characteristics and information about the most recent SARS‐CoV‐2 test were collected from medical records.

### 2.3 Air Sampling

Air was sampled from patient rooms at predefined distances of 1, 1–2, or 2–4 m from the patient's head. A liquid cyclone (Coriolis µ, Bertin Instruments, France) was used to collect air samples. The cyclone operated at 200 L/min for 10 min, with 15 mL of phosphate‐buffered saline solution as collection liquid, in single‐use collection vials.

### 2.4 Laboratory Analysis

The collected air samples were concentrated using Amicon Ultra‐15 centrifugal filter units (50‐kDa cutoff; Merck Millipore KGaA) to a final volume of 140 µL, which was used for RNA extraction using the QIAamp viral RNA mini kit (Qiagen, Germany). Reverse transcription–quantitative PCR (RT‐qPCR) was performed, with two replicates, using qPCRBIO Probe 1‐Step Virus Detect kit (PCR Biosystems) with primers and probes targeting the SARS‐CoV‐2 E‐gene, as previously described [12]. Serial dilutions of an in‐house quantified DNA amplicon containing the E and N gene (see Supporting Information S1: Figure S1). Samples with a Ct‐value < 40.5 were considered positive. Negative controls were collected with respect to air sampling, sample handling, and RT‐qPCR.

### 2.5 Statistical Analysis

Logistic regression and Chi‐squared test were used to explore differences between air samples collected from patients with pre‐Delta and Omicron infections. STATA SE/15.1 (StataCorp, College Station, TX, USA) was used for all statistical analyses.

## 3 Results

Patient and environmental characteristics of the rooms where the air samples were collected are presented in Table 1, with a comparison between the initial wave with ancestral virus and Alpha variants and the later Omicron wave. In total, 75 air samples were collected during the Omicron period, of which 4 (5%) were positive for SARS‐CoV‐2 RNA (Table 2). All positive air samples had high Ct‐values (≥ 39) close to detection limit, indicating low concentrations. Air samples were collected from rooms with 43 unique patients. A median of two (range: 1–4) samples were collected from each patient's room air. Ten of the patients received high flow nasal oxygen, two were treated with non‐invasive ventilation and two were also treated with nebulizer during air sample collection. Eleven of the 43 patients were considered immunocompromised. Seven had ongoing treatment with monoclonal antibodies and/or remdesivir. Infecting subtype was established in 30 of the 43 patients, with BA.2 and BA.1 in 26 (87%) and 4 (13%), respectively. The four positive air samples were collected from three different patients, two patients with BA.2, and one without subtype analysis. The results of the SARS‐CoV‐2 PCR analyses of patient room air from the Omicron period and the initial period are presented in Table 2.

**Table 1:** Air samples collected in patient rooms with Omicron or pre‐Delta SARS‐CoV‐2 variants and patient and environmental factors.

		Omicron	Pre‐Delta*	*p*
	Total samples	75	120	
Patient	Age, median (IQR)	78 (68–84)	61 (55–77)	< 0.05
	Sex male %	61%	70%	0.2
	Days from symptom onset, median (IQR)	9 (5–14)	12 (9–16)	0.6
	Days from admission, median (IQR)	4 (2–7)	4 (2–8)	0.3
	Patient Ct‐values within 5 days^1^, median (IQR)	20 (18–24) (*n* = 60)	24.2 (21–30) (*n* = 83)	< 0.05
	Previous SARS‐CoV‐2 immunization (2–3 vaccine doses)	55 (77%)	0 (0%)	< 0.05
	Antiviral treatment^2^	15 (20%)	0 (0%)	< 0.05
	Sampling distance			
Environment	< 1 m	26 (35%)	50 (43%)	< 0.05
	1–2 m	24 (32%)	50 (43%)	
	> 2 m	24 (32%)	16 (14%)	
	Room ventilation			
	Normal	31 (41%)	39 (33%)	0.2
	Enhanced	44 (59%)	81 (67%)	
	Single room	70 (93%)	109 (91%)	0.5
AGP	HFNO^3^	16 (21%)	43 (36%)	< 0.05
	NIV^4^	4 (5%)	4 (3%)	0.5
	Nebulizer treatment	4 (5%)	7 (6%)	0.9
	PEP training^5^	0	11 (9%)	< 0.05
	Any potential AGP^6^	22 (29%)	65 (54%)	< 0.05

*Note:* *Data extracted from Thuresson et al. [11].

^1^ Ct‐value from a recent (within 5 days from air sampling) upper airway SARS‐CoV‐2 PCR.

^2^ Including monoclonal antibody treatment and/or remdesivir.

^3^ High flow nasal oxygen.

^4^ Non‐invasive ventilation, with CPAP or BiPAP.

^5^ Positive expiratory pressure training, with a PEP‐flute.

^6^ Aerosol‐generating procedures (proposed), here including HFNC, NIV, nebulizer treatment, PEP‐training.

**Table 2:** SARS‐CoV‐2 RNA positive air samples collected in patient rooms with Omicron or pre‐Delta variants.

		Omicron: Positive/total air samples	% pos	Pre‐Delta: Positive/total air samples*	% pos
	Total samples	4/75	5%	14/120	12%
	Age (years)				
Patient and COVID‐19	< 55	2/10	20%	4/31	13%
	55–75	1/23	4%	5/49	10%
	> 75	1/42	2%	5/40	13%
	Sex				
	Male	3/46	9%	10/84	11%
	Female	1/29	3%	4/36	13%
	Days from symptom onset				
	1–7	3/36	7%	3/27	11%
	8–14	1/24	4%	9/55	16%
	> 15	0/15	0%	2/38	5%
	Patient Ct‐values within 5 days^1^				
	< 25	4/48	8%	8/46	17%
	> 25	0/12	0%	1/37	3%
	Immunization status (vaccine doses)				
	2–3	3/55	5%	0/0	
	0	1/16	13%	14/120	12%
	Antiviral treatment^2^				
	Yes	2/15	13%	0/0	
	No	2/60	3%	14/120	12%
	Sampling distance (m)				
Environment	< 1	1/26	4%	8/50	16%
	1–2	2/24	6%	3/50	6%
	> 2	1/24	4%	2/16	13%
	Room ventilation				
	Normal	2/31	6%	8/39	21%
	Enhanced	2/44	5%	6/81	7%
	Single room				
	Yes	4/70	6%	14/109	13%
	No	0/5	0%	0/11	0%
	HFNO^3^				
AGP	Yes	0/16	0%	3/43	7%
	No	4/59	7%	11/77	14%
	NIV^4^				
	Yes	1/4	25%	0/4	0%
	No	3/71	4%	14/116	12%
	Nebulizer treatment				
	Yes	1/4	25%	0/7	0%
	No	3/71	4%	14/113	10%
	PEP‐training^5^				
	Yes	0/0		4/11	36%
	No	4/75	5%	10/109	9%
	Any potential AGP^6^				
	Yes	1/22	5%	7/65	11%
	No	3/53	6%	7/55	13%

*Note:* *Data extracted from Thuresson et al. [11].

^1^ Ct‐value from a recent (within 5 days from air sampling) upper airway SARS‐CoV‐2 PCR.

^2^ Including monoclonal antibody treatment and/or remdesivir.

^3^ High flow nasal oxygen.

^4^ Non‐invasive ventilation, with CPAP or BiPAP.

^5^ Positive expiratory pressure training, with a PEP‐flute.

^6^ Aerosol‐generating procedures (proposed), here including HFNC, NIV, nebulizer treatment, PEP‐training.

In comparison with the results from our previous study in the same settings, no differences were found between proportions of SARS‐CoV‐2 positive air samples during the Omicron BA.2 wave and the primary wave of pre‐Delta SARS‐CoV‐2, even when adjusting for patient characteristics and setting (see Table 3). At patient level, 3 of 43 (7%) patients had at least one positive air sample during the Omicron period, compared to 10 of 60 (17%) during the initial period (*p* = 0.14).

**Table 3:** Adjusted odds ratio for an air sample to be positive for SARS CoV‐2 comparing Omicron vs. ancestral virus adjusted for confounding variables.

	Odds ratio	Confidence interval 95%	*p*‐value
Total samples	0.43	0.13–1.3	0.15
Adjusted for			
Age (years)	0.51	0.16–1.7	0.27
Days from symptom onset (days)	0.40	0.12–1.3	0.12
Patient Ct‐values within 5 days^1^	0.39	0.11–1.4	0.15
Sampling distance	0.48	0.15–1.6	0.22
Room ventilation	0.38	0.12–1.2	0.11

^1^ Ct‐value from a recent (within 5 days from air sampling) upper airway SARS‐CoV‐2 PCR.

In unadjusted analysis with all air samples from the previous study, which also included 111 air samples from ICU gave similar results for Omicron versus pre‐Delta to be positive in air (OR = 0.54, 95% CI = 0.18–1.6, *p* = 0.27).

## 4 Discussion

In this study, repeating air sample collection from the rooms of patients with ongoing SARS‐CoV‐2 infection, no difference in prevalence of SARS‐CoV‐2 RNA in the air between Omicron and pre‐Delta virus variants could be found. The tendency was that airborne SARS‐CoV‐2 RNA was more rarely occurring during the Omicron period, contrary to pre‐study perception.

This study is, to our knowledge, the largest investigation of Omicron in patient room air. However, the still limited number of collected air samples and few positive outcomes makes it difficult to draw firm conclusions when trying to compare the results with the initial period. The current study had the statistical power (0.8, alpha = 0.05) to detect a significant difference between the virus variants if the proportion of Omicron‐positive air samples was less than 2% or higher than 29% in an unadjusted analysis. This study found a lower prevalence of Omicron RNA in air compared to previous studies. Ong et al. [13] reported at least one positive air sample from 21 out of 38 (55%) Omicron‐infected patients, which was more frequent than for the Delta variant. Compared to the present study, Ong et al. [13] collected air closer to the patient's head and a few days earlier since symptom onset.

One possible explanation to our results of low rate of positive air samples in patient rooms during the Omicron period, contrasting to a high reported transmissibility, might be a different viral shedding kinetic to ancestral variants, as Omicron may have a higher early, but shorter shedding as indicated also by the high secondary attack rate but shorter infection serial interval [14, 15, 16]. However, due to changes in case population and situation between the initial pre‐Delta period and the Omicron period, including sampling distances, room ventilation, need for AGP, immunization and treatment, conclusions around differences in viral shedding could not be drawn from our study since a standardized and fully adjusted comparison of variants was not possible.

Our results still indicate that any increased hospital transmission during the Omicron wave was not primarily driven by hospitalized patients diagnosed with COVID‐19 as primary diagnosis, but instead, more likely, by patients and health‐care workers with a recent undiagnosed infection.

Our study has some additional limitations. The number of included patients and air samples collected was too small to permit multivariable analyses of any association to positive air samples and other potentially important variables, such as vaccination status, antiviral treatment, and immunosuppression. No typing of the virus strains was performed during the initial wave, but only a small proportion (~10%) could have been collected from Alpha variant infected patients and the Delta variant was introduced later. We used different primers in the RT‐qPCR compared to the previous study, though these two different primers had comparable sensitivity and yielded comparable Ct‐values (data not shown).

In conclusion, the occurrence of SARS‐CoV‐2 positive air in patient rooms of Omicron infected patients seems equal to or lower compared to the pre‐Delta variants. This suggest other factors as the main drivers behind any increased hospital transmission during the Omicron wave.

## Author Contributions

**Carl‐Johan Fraenkel:** conceptualization (lead), formal analysis, investigation (lead), writing–original draft preparation, writing–reviewing and editing (equal). **Sara Thuresson:** investigation, writing–reviewing and editing (equal). **Patrik Medstrand:** methodology, resources (lead), writing–reviewing and editing (equal). **Malin Alsved:** investigation, resources (supporting), funding, writing–reviewing and editing (equal). **Jakob Löndahl:** conceptualization (supporting) supervision, funding, writing– reviewing and editing (equal).

## Ethics Statement

The study was approved by the National Ethical Review Board in Sweden (project number 2020‐01396).

## Consent

Oral consent was given when possible, no formal written consent was collected.

## Conflicts of Interest

The authors declare no conflicts of interest.

## Permission to Reproduce Material From Other Sources

The manuscript contains original new data. As to the new data, a comparator extracted data, previously published, has been used. The current authors are the copyright owners and authors also of those previously published data. This is clearly disclosed in the manuscript.

## Supporting information

Supporting information.

## Data Availability

The data that support the findings of this study are available on request from the corresponding author.

## References

[1] P. V. Markov , M. Ghafari , M. Beer , et al., “The Evolution of SARS‐CoV‐2,” Nature Reviews Microbiology 21 (2023): 361–379. 37020110 10.1038/s41579-023-00878-2

[2] J. R. Port , C. K. Yinda , V. A. Avanzato , et al., “Increased Small Particle Aerosol Transmission of B.1.1.7 Compared With SARS‐CoV‐2 Lineage A In Vivo,” Nature Microbiology 7, no. 2 (2022): 213–223. 10.1038/s41564-021-01047-y PMC11218742 35017676

[3] O. O. Adenaiye , J. Lai , P. J. B. de Mesquita, , et al., “Infectious Severe Acute Respiratory Syndrome Coronavirus 2 (SARS‐CoV‐2) in Exhaled Aerosols and Efficacy of Masks During Early Mild Infection,” Clinical Infectious Diseases 75, no. 1 (2022): e241–e248, 10.1093/cid/ciab797. 34519774 PMC8522431

[4] J. Lai , K. K. Coleman , S. H. S. Tai , et al., “Exhaled Breath Aerosol Shedding of Highly Transmissible Versus Prior Severe Acute Respiratory Syndrome Coronavirus 2 Variants,” Clinical Infectious Diseases 76, no. 5 (2023): 786–794. 36285523 10.1093/cid/ciac846 PMC9620356

[5] M. Klompas , M. C. Pandolfi , A. B. Nisar , M. A. Baker , and C. Rhee , “Association of Omicron vs Wild‐Type SARS‐CoV‐2 Variants With Hospital‐Onset SARS‐CoV‐2 Infections in a US Regional Hospital System,” Journal of the American Medical Association 328, no. 3 (2022): 296–298. 35704347 10.1001/jama.2022.9609 PMC9201738

[6] J. Raymenants , W. Duthoo , T. Stakenborg , et al., “Exhaled Breath SARS‐CoV‐2 Shedding Patterns Across Variants of Concern,” International Journal of Infectious Diseases 123 (2022): 25–33. 35932968 10.1016/j.ijid.2022.07.069 PMC9349369

[7] J. Zheng , Z. Wang , J. Li , et al., “High Amounts of SARS‐CoV‐2 in Aerosols Exhaled by Patients With Omicron Variant Infection,” Journal of Infection 84, no. 6 (2022): e126–e128. 35183607 10.1016/j.jinf.2022.02.015 PMC8852223

[8] K. S. Tan , S. W. X. Ong , M. H. Koh , et al., “SARS‐CoV‐2 Omicron Variant Shedding During Respiratory Activities,” International Journal of Infectious Diseases 131 (2023): 19–25. 36948451 10.1016/j.ijid.2023.03.029 PMC10028358

[9] K. P. Y. Hui , J. C. W. Ho , M. Cheung , et al., “SARS‐CoV‐2 Omicron Variant Replication in Human Bronchus and Lung Ex Vivo,” Nature 603, no. 7902 (2022): 715–720. 35104836 10.1038/s41586-022-04479-6

[10] M. Klompas and A. Karan , “Preventing SARS‐CoV‐2 Transmission in Health Care Settings in the Context of the Omicron Variant,” Journal of the American Medical Association 327, no. 7 (2022): 619–620. 35072715 10.1001/jama.2022.0262

[11] S. Thuresson , C. J. Fraenkel , S. Sasinovich , et al., “Airborne Severe Acute Respiratory Syndrome Coronavirus 2 (SARS‐CoV‐2) in Hospitals: Effects of Aerosol‐Generating Procedures, Hepa‐Filtration Units, Patient Viral Load, and Physical Distance,” Clinical Infectious Diseases 75, no. 1 (2022): e89–e96. 35226740 10.1093/cid/ciac161 PMC9383519

[12] V. M. Corman , O. Landt , M. Kaiser , et al., “Detection of 2019 Novel Coronavirus (2019‐nCoV) by Real‐Time RT‐PCR,” Eurosurveillance 25, no. 3 (2020): 2000045, 10.2807/1560-7917.ES.2020.25.3.2000045. 31992387 PMC6988269

[13] D. S. Y. Ong , P. de Man , T. Verhagen , et al., “Airborne Virus Shedding of the Alpha, Delta, Omicron SARS‐CoV‐2 Variants and Influenza Virus in Hospitalized Patients,” Journal of Medical Virology 95, no. 4 (2023): e28748. 37185846 10.1002/jmv.28748

[14] O. Puhach , B. Meyer , and I. Eckerle , “SARS‐CoV‐2 Viral Load and Shedding Kinetics,” Nature Reviews Microbiology 21, no. 3 (2023): 147–161. 36460930 10.1038/s41579-022-00822-w PMC9716513

[15] Z. J. Madewell , Y. Yang , I. M. Longini, Jr. , M. E. Halloran , A. Vespignani , and N. E. Dean , “Rapid Review and Meta‐Analysis of Serial Intervals for SARS‐CoV‐2 Delta and Omicron Variants,” BMC Infectious Diseases 23, no. 1 (2023): 429. 37365505 10.1186/s12879-023-08407-5 PMC10291789

[16] S. W. Kang , J. Y. Kim , H. Park , et al., “Comparison of Secondary Attack Rate and Viable Virus Shedding Between Patients With SARS‐CoV‐2 Delta and Omicron Variants: A Prospective Cohort Study,” Journal of Medical Virology 95, no. 1 (2023): e28369. 36458559 10.1002/jmv.28369 PMC9877691

