# Supplementary material for: Comparison of Airborne SARS‐CoV‐2 Omicron and Pre‐Delta Variants Around Infected Patients
Source: J Med Virol. 2025 Feb 20;97(2):e70258. doi: 10.1002/jmv.70258 (PMC11841929; doi:10.1002/jmv.70258)
Supplement: Supplementary file 1 — Supporting information. [file JMV-97-e70258-s001.docx]

**Supplement**

**Omicron vs ancestral SARS-CoV-2 variants in hospital air**

**Suppl. Figure 1.** Performance of SARS-CoV-2 PCR with N- and E-gene primers in different virus dilution.
